# Youth Perspectives on ‘Highly Personalised and Measurement‐Based Care’: Qualitative Co‐Design of Education Materials

**DOI:** 10.1111/hex.14137

**Published:** 2024-07-08

**Authors:** Sarah McKenna, Alexis Hutcheon, Carla Gorban, Yun Song, Elizabeth Scott, Ian Hickie

**Affiliations:** ^1^ The Brain and Mind Centre The University of Sydney Camperdown New South Wales Australia

## Abstract

**Objectives:**

Despite high levels of mental ill‐health amongst young people (aged 15–30), this group demonstrates low help‐seeking and high drop‐out from mental health services (MHS). Whilst shared decision‐making can assist people in receiving appropriate and effective health care, young people frequently report that they do not feel involved in treatment decisions. The current study focused on co‐design of a clinical education and participant information programme for the Brain and Mind Centre Youth Model of Care. This model, which articulates a youth‐focused form of highly personalised and measurement‐based care, is designed to promote shared decision‐making between young people and clinical service providers.

**Methods:**

We conducted workshops with 24 young people (16–31; *M*_Age_ = 21.5) who had accessed mental health services. Participants were asked what advice they would give to young people entering services, before giving advice on existing materials. Workshops were conducted and transcripts were coded using thematic analysis by two lived experience researchers and a clinical researcher.

**Results:**

Young people found it empowering to be educated on transdiagnostic models of mental illness, namely clinical staging, which gives them a better understanding of why certain treatments may be inappropriate and ineffective, and thus reduce self‐blame. Similarly, young people had limited knowledge of links between mental health and other life domains and found it helpful to be educated on multidisciplinary treatment options. Measurement‐based care was seen as an important method of improving shared decision‐making between young people and health professionals; however, to facilitate shared decision‐making, young people also wanted better information on their rights in care and more support to share their expertise in their own needs, values and treatment preferences.

**Conclusions:**

These findings will inform the delivery of the further development and implementation of a youth‐specific clinical education and participant information programme for the BMC Youth Model.

**Patient or Public Contribution:**

Workshops were facilitated by researchers with lived expertise in mental ill‐health (A.H. and/or C.G.) and a clinical researcher (who has expertise as an academic and a clinical psychologist). A.H. and C.G. were also involved in conceptualisation, analysis, interpretation, review and editing of this paper.

## 1 INTRODUCTION

Around 75% of severe mental disorders emerge during adolescence and young adulthood [1, 2, 3, 4, 5]. Moreover, the burden of mental ill‐health is greatest between the ages of 15 and 25 as compared to any other stage of life because of the complex biological and psychosocial changes that occur during this period [6, 7]. Yet, despite the urgency of identifying and responding to emerging disorders, current diagnostic and assessment frameworks struggle to predict the severity and complexity of young people's needs so that they can receive appropriate treatments early [8, 9, 10, 11, 12]. Additionally, early intervention services are struggling to provide the multidisciplinary treatments needed to address this complexity. Over 80% of activities in these services are related to assessment and mental health therapies, and less than 5% of activity is focused on drug and alcohol use or educational and occupational functioning [2]. Whilst urgent reforms are needed, mental health professionals (MHPs) and services have been slow to adopt new technologies and practices that are likely to lead to rapid improvements [13, 14, 15, 16, 17]. It is thus important for change to occur in partnership with young people themselves, ensuring that those most affected are empowered to make shared decisions with health professionals about improving their treatments.

The Brain and Mind Centre (BMC) has developed a Youth Model of Care, which explicitly aims to prevent the progression to more severe mental disorders in young people through highly personalised and measurement‐based treatments [4, 18]. A key aspect of this model is the allocation of care based on clinical staging, which places young people on a continuum (reflecting the underlying pathophysiological mechanisms and illness trajectories) based on the risk of progression to more severe disorders so that different levels of care can be assigned to those with different risk factors and levels of impairment [9, 10, 11, 19]. Our model also argues that to accurately stage care for young people, assessment and treatment approaches need to address pathophysiological mechanisms that underlie trajectories to severe mental disorders and emphasise multidisciplinary care options that meet the complexity of young people's needs [20, 21, 22, 23, 24]. Moreover, in line with calls from leading health organisations, our model argues that technology is needed to improve routine outcome monitoring (ROM) and feedback so that treatments are more responsive and personalised to individuals' needs [4, 21, 25, 26, 27]. Taken together, implementing highly personalised and measurement‐based care in youth services can radically transform their capacity to identify and respond to emerging severe mental disorders.

These principles are grounded in current evidence about the emergence of severe mental disorders and effective early treatment. Longitudinal research demonstrates that, in the early stages of major mood and psychotic disorders, symptoms are largely transdiagnostic, with most individuals experiencing a mix of non‐specific symptoms, such as anxiety, depression, sleep disturbance, withdrawal and apathy [28, 29]. For some, these symptoms are transient and intermittent, for others they become worse over time evolving into severe mental disorders [4]. There is also evidence that those at later stages of illness have more complex needs, such as higher rates of not being in school, education or work, requiring multidisciplinary treatments [1, 4, 20]. Thus, the transdiagnostic clinical staging approach better characterises those in the early stages of severe mental disorders [30, 31].

Meanwhile, there is also strong support for the efficacy of ROM and the benefits of this approach are enhanced when health information technologies are used. Large meta‐analyses have found that ROM can reduce deterioration rates and nearly double clinically significant/reliable change rates in clients who are predicted to have a poor outcome [14, 16, 32, 33]. Despite this, health services have been slow to implement ROM technologies. A national survey in the United States found that only 13.9% of clinicians reported using standardised progress measures at least monthly and 61.7% never used them [13]. Similar usage rates have been found in smaller studies in Australia [34]. As such, alternative strategies are needed to improve the transformation of services and these strategies should include empowering young people to make shared decisions with health professionals about highly personalised and measurement‐based treatments.

When young people are involved in shared decision‐making they are significantly less likely to disengage from care, are more satisfied with their treatment and experience better outcomes, suggesting they need to be educated on the principles of highly personalised and measurement‐based care [35, 36]. Shared decision‐making refers to a collaborative process by which clients and MHPs make joint treatment decisions based on the MHP's expert knowledge of mental health treatments and the individual's expert knowledge of their own values, needs and experiences [35]. This is considered to be the right of all individuals seeking care by the United Nations and many other professional health organisations [37]. Yet, current data suggests that young people generally do not perceive they are involved in mental health treatment decisions. Guided Self‐Determination theory argues that this is most often caused by poor communication between health professionals and clients [38, 39]. Often, MHPs are focused on symptom reduction whereas young people are focused on returning to ‘normal life’. At the same time, young people tend to believe they are not capable of contributing to healthcare discussions and so may not raise concerns when treatment goals do not address problems that are most important to them e.g., when MHPs focus on managing unhelpful thinking patterns rather than returning to school [38, 39]. Thus, shared decision‐making in youth mental health services can be improved by increasing young people's knowledge of mental health and treatment processes, so they are better able to discuss their needs.

Accordingly, lived expertise researchers on our team developed education and training materials that could be used to empower young people to access highly personalised and measurement‐based care. Education and training programmes have commonly been used to improve youth help‐seeking and to improve young people's perceived involvement in shared‐decision making, with positive effects [35, 36, 40, 41]. The current study qualitatively explored whether these training materials were seen as valuable by young people and would assist them in seeking highly personalised and measurement‐based treatments.

## 2 Methods

### 2.1 Settings and Participants

Six workshops were held in November 2022 with 24 young people aged 16–31 who had previously sought professional treatments for mental health‐related problems. Of the 24 young people, 80% were female and the mean age was 21.5. Between three and seven participants attended each workshop of which four were face‐to‐face only and two were held entirely on Zoom (there were no hybrid workshops). Workshops were facilitated by a researcher with lived expertise in mental ill‐health and a clinical researcher (who was an academic and clinical psychologist).

We employed purposive sampling to recruit young people with existing experience of accessing mental health services, as they were most likely to be aware of challenges to accessing care and to know how these barriers had been overcome in their own treatment. Participants were excluded if they were over the age of 31, if they had not previously accessed a mental health service and if they were non‐English speaking. Young people were recruited through flyers and online advertisements at health services that had existing strong partnerships with our team (including headspace services in Camperdown, Coffs Harbor, Bondi Junction and Port Macquarie, The University of Sydney student counselling services, and a private multidisciplinary clinic in Sydney). Three of these services were located in urban areas with high socioeconomic advantage whilst two of these services were located in regional areas with medium to low socioeconomic advantage [42]. Advertising materials provided QR codes for young people to access an information sheet and provide consent to participate.

### 2.2 Data Collection

Participants were first asked to reflect on “what advice would you give to young people seeking care for the first time?” to explore participants' existing beliefs and attitudes about accessing mental health treatments. Participants were asked to submit answers anonymously via a poll and then asked to discuss the themes that they saw as most important during a 40 min group discussion. Subsequently, participants were given a short presentation using education materials that had been designed by lived expertise researchers on principles of the BMC Youth Model, including shared decision‐making, levels of care appropriate for different needs, multidisciplinary treatment options and technology‐assisted MBC. After each topic was presented, a group discussion was held during which participants were asked to reflect on whether they found this content valuable and how it could be improved. Workshops were audio‐recorded and then transcribed by members of the research team (S.M., A.H. and C.G.). Transcriptions were deidentified to protect participants' confidentiality. All recordings and transcriptions were stored in a secure data store provided by The University of Sydney.

### 2.3 Data Analysis

Our analysis of workshop transcripts followed the procedures for thematic analysis as this approach facilitates both inductive (top‐down) and theory‐driven data analyses [43]. Our analysis was driven by what was in the data and by concepts and ideas brought by researchers. Audio recordings of workshops were transcribed and read multiple times by a clinical researcher (S.M.) who was the primary coder and two lived experience researchers (A.H. and C.G.) who were secondary coders, to establish common patterns across workshops. Preliminary analyses were conducted by these three researchers and focused on identifying common themes across workshops. Preliminary codes were then shared and discussed to establish initial themes. Following this, existing theories and research on highly personalised, measurement‐based care and shared decision‐making were used by the primary coder to deductively organise these themes under four broad topic areas aligned with existing frameworks. Subsequently, a second round of coding (involving all three coders) was conducted to establish broader patterns of meaning within each topic area. Themes from this round were again discussed amongst the coding team and refined.

Principles of constructionist theory grounded our analysis, which argues that all knowledge is constructed through the experiences and subjectivities that researchers bring to the data. S.M. is a clinical psychologist and is experienced in cognitive‐behavioural therapies that emphasise complex interrelationships between attitudes, experiences and behaviours. A.H. and C.G. are researchers with lived expertise who know other contextual factors that influence how individuals experience mental health treatments. These pre‐existing subjectivities shaped our interpretation of the data.

### 2.4 Involvement of Lived Expertise

We sought to include the voices of young people with lived expertise in seeking mental health treatments in this process to ensure that our work is closely aligned with the values and preferences of the population we are seeking to help [44, 45, 46]. Two researchers with lived experience, who are permanently employed by our team, designed educational materials and resources for young people, and assisted with recruiting participants, planning and facilitating co‐design workshops and interpreting data.

## 3 Results

### 3.1 Translating the BMC Youth Model of Care

#### 3.1.1 Staging Care Options for Mental Ill‐Health

Overall, participants reported that they had a poor understanding of the types of care available to them and did not feel confident or knowledgeable enough to seek different treatment options (such as alternative forms of evidence‐based therapies or specialist health professionals) even if they were not benefitting from care, preferring to drop out of care prematurely. Thus, materials that could encourage young people to seek care early, and clearly explain the types of care available based on type and severity of need were thought to be highly valuable. Participants were highly interested in gathering more information about the nature of their illness and their likelihood of progressing to more severe mental disorders. This information reduced the shame associated with not getting better from inappropriate care and helped them understand what care options to seek out. As shown below, participants frequently discussed feeling relieved when they had been told that their current level of care was not adequate for their needs, as it had prevented them from personalising their lack of progress and thus reduced self‐blame.

I think it would make sense because I wasn't getting the right care … but if I had known that, yeah, there were different levels of care, and that I needed a higher level of care, that would have been beneficial.

(Female, 19)

When my attempt happened, my psychiatrist, asked ‘do you have a psychologist with you?’ ‘Yeah, I said’ … he's like, ‘oh thank God, you need it’. You know, like, ‘I know, I'm not enough’. And that felt really reassuring that my psychiatrist was like, ‘I'm not enough, you need a therapist’.

(Female, 21)

As discussed in these excerpts, young people had found it reassuring to be told by health professionals that the reason they were not progressing or getting worse was because they needed a higher level of care. In a similar vein, one young person above who had struggled to benefit from care in the past, and had experienced feelings of hopelessness and shame because of it, reported feeling relieved after being given our training.

Even so, it was consistently emphasised that education materials should be cautious in how they use the term ‘stages’ in relation to mental ill‐health. As explained in the excerpt below, participants frequently reported that they had delayed accessing treatment because they did not feel ‘serious enough’ and believed that language such as ‘staging’ may prompt young people to compare the severity of their illness with others, which could deter them from seeking care or may encourage them to become more ‘serious’.

I'm just mindful that for some young people [discussing stages of care] might actually feel really dismissing, really invalidating and might actually make them like, not want to engage, because they feel like they're just gonna get thrown out.

(Female, 26)

There's a part of depression for some people, where you kind of constantly compare yourselves to others … there's always a part of me that's like, let's just get worse and worse and see where it goes … So I think it more has to be just a general information, like not different levels and stuff.

(Female, 22)

As shown here, whilst participants wanted more information about the nature of their illness and their illness trajectory, they also emphasised that young people should be discouraged from comparing their illness trajectory to others as this may be ‘invalidating’, meaning language such as ‘levels’ or ‘stages’ could be unhelpful.

One participant neatly summarised this conflict. They (Female, 21) explained ‘you want to communicate to people that what you're going through it's not nothing, and it should be addressed regardless …’, in other words, this participant saw a risk that young people assigned to earlier stages may see themselves as not serious enough for care. Yet, this participant also acknowledged ‘there are definitely levels of severity and [it's] important to pinpoint [the stage] because if you don't know where to pinpoint that I'm not sure how you actually go about solving your problem?’. In other words, young people would benefit from a better understanding of their needs through education on transdiagnostic clinical staging; however, education materials must strongly emphasise that the intention of such models is for young people to seek care as early as possible to prevent progression to later stages of illness.

#### 3.1.2 Multidisciplinary Assessment and Care

Multidiscinary approaches to metnal health care are reccomended for youth populations (under 25) give the complex impacts of metnal disorders on all aspects of life, such as physical health, functioning, and social connectedness. Yet most participants reported that their MHPs were reluctant to discuss needs other than mental health symptoms, even when participants felt that other aspects of their lives were highly interconnected with their mental ill‐health. For example, as shown below, several young people in our workshops also had physical disabilities and appeared frustrated that MHPs had often not asked questions about these difficulties or had even declined to work on these domains because they were outside their scope of practice.

I am a Type One Diabetic and I felt that my therapist did not understand that well. And I found it hard, because I was like, not in a position of power in that dynamic …, to advocate for myself, and just let them know how important it was, to me to have this other area addressed.

(Male, 19)

I think in my previous experience, sometimes therapists can get stuck on one of these sections instead of realizing that they all you know, merge into each other and that one affects the other. So like I really appreciate this model because I really wish that, I [had] known about that sort of thing sooner I guess.

(Female, 19)

As shown here, participants tended to agree that it would be empowering for young people to be given better education about the links between mental health and other life domains, such as physical health and functioning. As expressed above, participants believed that MHPs often did not ‘know how important it was’ to address these domains and wanted young people to be better able to ‘advocate’ for their needs.

It should be noted that participants did not expect MHPs to be able to address all their needs. Rather, participants reported being ignorant about links between mental health and other life domains when first accessing care and believed that psychoeducation on these relationships would empower young people to have these needs addressed earlier in care; whether by MHPs themselves or through referral pathways. This conversation was linked to earlier comments about the value of understanding different types of care. Young people found it a relief to know that individual clinicians could not address all their needs and that they may be able to seek help from other health professionals if they found they were not progressing from one type of treatment.

Despite generally finding our resources to be valuable, participants also suggested improvements that they believed were important. Firstly, our resources did not include information about identity, yet across all workshops participants emphasised the importance of including this domain in our materials as it was seen to be strongly interconnected with participants' mental health. Aspects of identity that were most frequently discussed included culture, sexual orientation, gender and family background. Once again, it was seen as important to discuss the links between identity and mental health as participants believed their peers had poor knowledge of these relationships. Additionally, participants repeatedly asked for each domain to be represented as overlapping and interconnected, whereas the diagram we presented appeared to suggest that they could be seen as separate and treated individually. Again, this was linked to concerns that their peers had a poor understanding of how mental health is linked to other life domains.

#### 3.1.3 Technology‐Enhanced ROM

Overall, young people felt that technology‐enhanced MBC would facilitate shared decision‐making; however, they emphasised that they would need support from clinicians and health services to use this technology and would need to be educated on its value rather than being given ‘just another app’. One key benefit of such technology identified by participants was that they often forgot experiences that had occurred between appointments. This frequently led them to underreport symptoms, such as low mood or self‐harm, meaning that they were not properly addressed by MHPs. In fact, a common recommendation from participants during our initial open‐ended discussion, as shown below, was to encourage young people to prepare for sessions by listing symptoms and problems, as it was difficult to remember information about your mental health when you were in the room with your MHP.

I think it would be beneficial, especially if you aren't seeing your psychologist frequently, like, from personal experience, like with my mood fluctuates … I might go in to see my psychologist and be really good … and kind of like almost forget what has previously happened …

(Male, 27)

This participants suggests that young people commonly lack insight into their mental health status and tend to focus on how they are feeling that day, limiting the information they can give MHPs about their condition and potentially impacting the appropriateness of treatment. In a similar vein, another benefit of MBC identified by participants was that it could help young people to notice treatment progress.

I think it's important because, like oftentimes I felt like in therapy you kind of forget how far you've come after a certain while. And I think something like this could help you, like keep track in a more concrete way of the progress you're making.

(Female, 24)

Again, this young person is suggesting that they frequently ‘forget’ mood and other mental health symptoms over time, making it difficult to perceive when they have progressed from therapy and therefore whether they have found an effective treatment.

Overall, participants felt that it was important to be given tools they could use to gain more insight into their mental health, partly so that they could give their clinicians more accurate information to support their care, and so that they could better understand their own mental health profess. Whilst paper‐based tools, such as diaries, were also seen as helpful for this purpose, several participants commented that technology‐based measurement was more useful as they could store health information between episodes of care, reducing the burden of remembering their health history when accessing further treatments. As such, they appeared to value education on technology‐based tracking systems, such as apps or websites.

Yet, participants emphasised that these platforms would only be valuable if they were also used by clinicians. One participant (Female, 30) commented ‘I think it could be a really useful tool … [but] it wouldn't be useful unless your clinician was already trained in it, willing to use it, and thought it'd be good for you right’. Other young people who had used similar platforms were concerned that clinicians often did not discuss their data with them, suggesting that the onus is on young people to mention this information during sessions. Accordingly, whilst participants felt that it was important for young people to be educated on the value of recording outcomes, they emphasised that this would not lead to uptake of the technology unless clinicians demonstrated the value of the platform by discussing data in the session.

#### 3.1.4 Shared Decision‐Making

A key theme that was identified across all workshops, was helping young people to recognise their ‘expertise in themselves’ whilst also benefitting from professional experience. Being afraid to disagree with ‘experts’ was commonly seen as a barrier to shared decision‐making thus, as expressed below, all workshops included unprompted discussions about how to benefit from ‘expert’ knowledge whilst also feeling empowered to disagree.

Young people kind of get treated, like they don't know what's happening. So like psychoeducation, like 100% so important, but like also potentially the clarification that the people who have the most knowledge about their experiences is the person with the experience.

(Male, 27)

You need to advocate for yourself as hard as that is sometimes and knowing that, like, it's not always easy, but it's gonna be worth it in the long run.

(Male, 19)

These participants believe that young people find it ‘hard’ to voice their needs in therapy, partly because they ‘don't know what's happening’, and suggest that young people need to be given better information about how to share their knowledge of their experiences and advocate for themselves during treatment planning.

In addition, participants consistently discussed the importance of understanding the limits of shared decision‐making, both in response to our materials and unprompted at the start of the workshop. As expressed below, participants believed that this information could help young people understand when they were able to request alternative treatment options and when they may need to participate in treatments against their will.

I think even the explanation of that, and how that works can be something that's really helpful, so that you don't have a situation where like, a young person all of a sudden feels out of their depth and they don't have control … And then like, here is the situation in which we would take that out of your hands and this is what that would look like.

(Female, 22)

As shown, participants in our workshops believed that young people are often worried about ‘losing control’ of their treatment. Concerningly, this was often seen as a barrier to treatment and made young people hesitant to share severe symptoms, such as suicidality, with their health professionals. Thus, participants believed that giving young people a better understanding of the circumstances that may lead to involuntary care (i.e., forced hospital admissions) would improve their openness with health professionals and their engagement in treatment.

Likewise, a common concern throughout workshops was the ability of younger adolescents to be involved in decisions about their treatment. Participants consistently reported that they had felt forced to participate in therapy during childhood and early adolescence and wanted more guidance around legal and ethical guidelines that clinicians used to guide decision‐making when working with ‘minors’. Key issues that were commonly discussed across workshops included when it was possible for individuals to request their own Medicare card and how clinicians would balance differing opinions between individuals under the age of 15 (which is generally used by leading ethical bodies as the age at which individuals are capable of consenting to treatments independently) and their families. In sum, empowering young people to share their expertise in themselves and giving them a clearer understanding of their rights in care, is key to improving their confidence and capacity to be involved in treatment decisions.

## 4 Discussion

Our study sought to co‐design education materials for young people to explore how key aspects of highly personalised and measurement‐based care can be communicated. Overall, we found that young people did not have a strong understanding of the care options available to them, and did not understand how multidisciplinary treatments other than medications or psychological therapies could help to improve their mental health. Accordingly, they found it empowering to be educated on concepts, such as clinical staging and multidimensional care, and also thought these could reduce shame and hopelessness by helping young people to understand why early treatments were not working. We also found that shared decision‐making could be improved in early intervention services by educating young people on their rights in care, highlighting their expertise in themselves, and helping them gain better insights through health tracking technologies. As such, it appears that principles of highly personalised and measurement‐based care can empower young people to be more engaged in their mental health care.

As such, these findings significantly improve our capacity to engage young people in mental health services early in their illness journey. Young people in our research did not have a strong understanding of the links between mental health symptoms and other domains, such as physical health, and thought that they were not allowed to address these problems with their MHPs. This may help to explain recent findings from Australian youth mental health services that less than 5% of activity is related to alcohol and other drug services, education and training support or medical treatments [2]. Also, young people frequently delayed seeking treatments because they did not feel ‘serious enough’ or disengaged from treatments that were not working, rather than seeking other options [47, 48]. Again, this is consistent with the finding that over 50% of young people only attend one or two sessions at youth mental health services [2]. Our results suggest that a lack of knowledge about the availability and appropriateness of treatments may partly explain poor help‐seeking amongst young people. Future research should explore whether this knowledge can improve youth engagement in multidisciplinary mental health treatments.

These findings may also assist with implementing gold‐standard clinical practices in early intervention services. Young people were highly positive about practices such as ROM and discussed a number of important advantages of these approaches. In particular, they thought that more frequent tracking of their symptoms would help them to be involved in shared decision‐making, and to recognise progress from therapy. This is consistent with findings from previous meta‐analyses and reviews, and it was found that patients support ROM when its purpose is clear and used to empower clients to be more involved in their care [49]. Accordingly, it is concerning that only a small proportion of clinicians currently engage in measurement‐based care and successful implementation of ROM in health services has been limited [13, 33, 49]. Based on our results, health services should be encouraged to involve individuals with lived experience when they adopt new clinical practices so that MHPs better understand the impact on their clients. Implementation of novel clinical practices should also involve education for consumers on the potential benefits or risks. Having said this, longitudinal evaluation is needed to establish the true impact of our education programme on the implementation of highly personalised and measurement‐based care in services.

Despite these contributions, our study has several limitations that need to be discussed. First, whilst young people in our study provided valuable recommendations about empowering others to be involved in their treatments, we have not evaluated whether these recommendations can effectively improve shared decision‐making. As such, we aim to evaluate the extent to which our information materials improve young peoples' knowledge of treatments available to them, and whether this leads to more involvement in treatment decisions. A second important limitation is that we recruited young people who had already accessed youth mental health services, many of whom had accessed a number of different services over a number of years. Young people who have never accessed mental health services before may face unique barriers; for instance, low availability of services in their area, which were not addressed by our participants. Again, this creates a need to evaluate the effectiveness of our programme, particularly for those who have previously been reluctant or unable to access care.

Third, it should be noted that our sample was 80% female. Females in this age group are better at identifying psychological problems, have better awareness of mental health services available and barriers to care, and are also more likely to access mental health services [50]. This may help to explain why females were more likely to volunteer for our study, but may also suggest that there are further barriers to care that need to be explored in male populations and addressed in our education materials. Likewise, we excluded young people who could not speak English. There are increased rates of mental disorders amongst international student, immigrant and refugee populations in Australia, yet these groups are less likely to seek treatment due to factors, such as stigma, language or financial barriers or lack of knowledge about available treatments [51, 52, 53]. It is therefore important that future work address treatment engagement in these priority populations.

This study was focused on exploring how principles of highly personalised and measurement‐based care, as defined by the BMC's Youth Model of Care, could be translated for young people. Overall, participants reported that more education about principles of highly personalised and measurement‐based care could help to reduce shame when young people were not improving from treatments and encourage them to engage in more conversations with MHPs about appropriate treatment options. Thus, along with informing our own programme, the current study provides important insights for researchers and health professionals, which can help to improve engagement in youth mental health services.

## Author Contributions

**Sarah McKenna:** conceptualisation, methodology, investigation, formal analysis, writing–original draft, validation. **Alexis Hutcheon:** conceptualisation, investigation, formal analysis, data curation, writing–review and editing, visualisation, validation. **Carla Gorban:** conceptualisation, investigation, writing–review and editing, validation. **Yun Song:** conceptualisation, project administration, writing–review and editing, funding acquisition, supervision, resources. **Elizabeth Scott:** conceptualisation, writing–review and editing, funding acquisition, supervision, resources. **Ian Hickie:** conceptualisation, funding acquisition, writing–review and editing, supervision, resources.

## Ethics Statement

This research was approved by the University of Sydney Human Research Ethics Committee Protocol No.: 2021/277.

## Conflicts of Interest

I.H. is the Co‐Director, Health and Policy at the Brain and Mind Centre (BMC) University of Sydney. The BMC operates an early‐intervention youth services at Camperdown under contract to Headspace. He is the Chief Scientific Advisor to and a 3.2% equity shareholder in InnoWell Pty Ltd, which aims to transform mental health services through the use of innovative technologies. He is funded by an NHMRC Senior Principal Research Fellowship.

## Data Availability

The data that support the findings of this study are available on request from the corresponding author. The data are not publicly available due to privacy or ethical restrictions.
